# Open questions in transplutonium coordination chemistry

**DOI:** 10.1038/s42004-020-00338-5

**Published:** 2020-08-07

**Authors:** Korey P. Carter, Roger M. Pallares, Rebecca J. Abergel

**Affiliations:** 1 Chemical Sciences Division, Lawrence Berkeley National Laboratory, Berkeley, CA 94720 USA; 2 Department of Nuclear Engineering, University of California, Berkeley, CA 94709 USA

**Keywords:** Chemical bonding, Coordination chemistry

## Abstract

Over the past decade, momentous progress has been made in the characterization of late actinide compounds. Here the authors highlight how advances in spectroscopic and computational tools have developed our understanding of fundamental transplutonium bonding interactions, and discuss whether covalency and heterogeneity changes in 5f-orbital bonding could be harnessed in environmentally and industrially relevant systems.

Trends in structure and bonding that are well-defined elsewhere in the periodic table are underdeveloped in the actinide series, and in particular for the transplutonium elements, which are recognized for their scarcity and inherent radioactivity. Nevertheless, the last ten years have brought about a surge in interest in the chemistry of the later 5*f* elements as scientists have sought to better understand the distinctive chemical properties of the actinides series, which lie between those of the transition metals (for which valence 3*d*-, 4*d*-, and 5*d*-orbitals are available to form covalent bonds) and the lanthanides (for which ionic bonding predominates). Many of the chemical and physical properties of the transplutonium elements have not been characterized, even for those available in sufficient quantities for classical macroscopic experiments: americium (Am), curium (Cm), berkelium (Bk), and californium (Cf). Despite characterization limitations, each of these heavier actinides have found applied uses including as radioisotope thermoelectric generators^1,2^, as targets for superheavy element discovery^3^, and as neutron activation sources^4^.

Hampered by the limited capabilities of most scientific laboratories, current knowledge of transplutonium coordination chemistry had, until recently, either been extrapolated from lanthanide surrogates, or obtained through tracer techniques and in silico methods. Combined with increased access to transplutonium isotopes, large leaps in instrumentation development and data processing have collectively expanded the scope of what is possible to study. This resurgence was instigated in 2010 by Galbis et al.^5^ who combined extended X-ray absorption fine structure (EXAFS) spectroscopy with Monte Carlo simulations to resolve the solvation of Cf^3+^ ions, and experimentally confirm the actinide contraction extends to Cf. Since then, EXAFS has been applied to explore the solution chemistry of several transplutonium complexes, and significant deviations from predicted behavior, based on lanthanide results, have been observed^6–8^. For instance, our group investigated late actinide binding with the well-known chelator diethylenetriaminepentaacetic acid (DTPA), and found a larger than expected decrease in M–O bond distances at Cf (Fig. 1a, b)^6^. These results confirmed that M–O bonds in transplutonium-DTPA complexes likely feature some amount of heterogeneity, and the origin and extent of this covalency in metal-ligand bonding is a topic at the forefront of fundamental actinide chemistry.

**Fig. 1:**
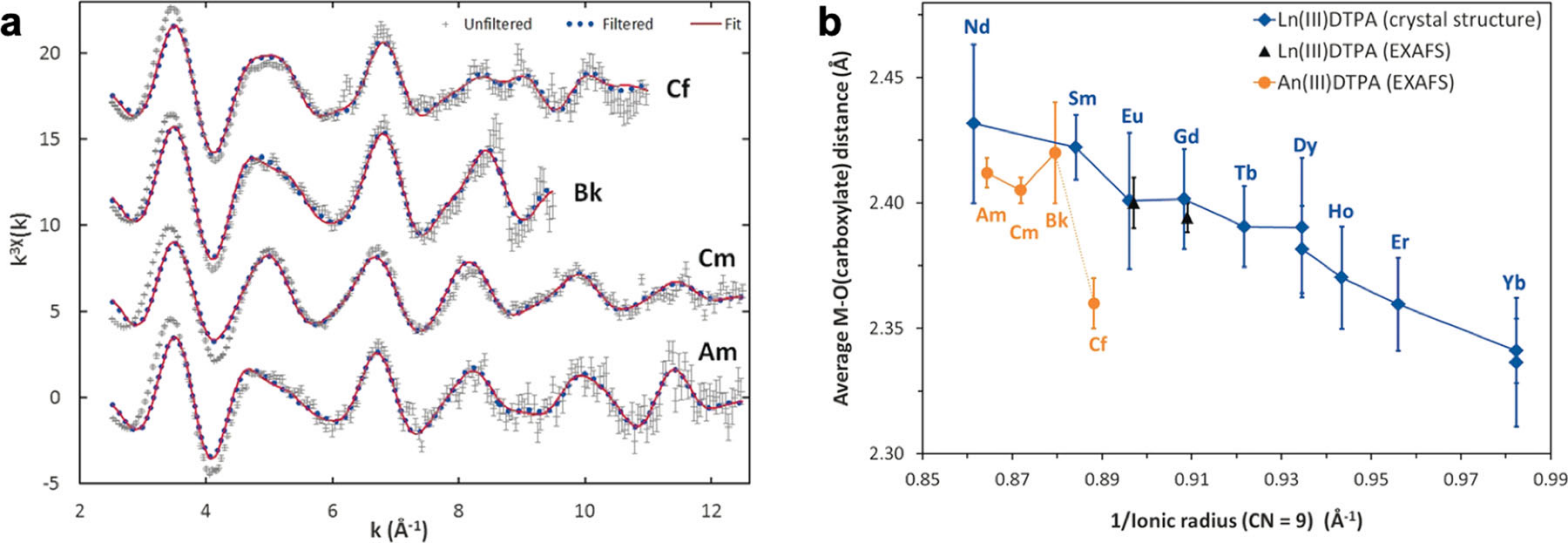
Structural features of An^III^-DTPA complexes probed using element-specific L_3_-edge X-ray absorption spectroscopy. **a** EXAFS data and fit. **b** Metal–oxygen bond lengths of lanthanide and actinide complexes with DTPA. Error bars correspond to the standard deviation among M–O bonds for a given crystal structure of EXAFS structural model. Adapted with permission from ref. ^6^. Copyright 2018 Wiley-VCH Verlag GmbH & Co.

In parallel, X-ray diffraction (XRD) techniques have advanced to the point where obtaining results with transplutonium complexes is feasible, which has proven valuable in improving our understanding of the role of valence orbitals in late actinide bonding. Pioneering work in this area was done by Albrecht-Schmitt et al. who synthesized and characterized single crystals of dipicolinate (DPA) and borate complexes with Bk^III^ and Cf^III^, which displayed unexpected evidence of covalency in Bk- and Cf–O bonds in corresponding magnetic measurements^9–11^. More recent efforts from Bart et al. and Wilson and colleagues; however, have demonstrated the higher degree of covalent interactions in actinide complexes may be ligand dependent, as transplutonium complexes with dioxophenoxazine (DOPO) and thiocyanate ligands feature M–O bonds that are primarily ionic, as probed via electronic structure calculations and Raman spectroscopy, respectively^12,13^.

To better understand the extent and impact of covalency on transplutonium compounds, actinide chemists have expanded their structural and spectroscopic toolbox, thereby taking advantage of techniques that offer unique characterization capabilities. We have taken inspiration from biological systems, using macromolecular crystallography to study the binding of late actinide small molecule complexes by a mammalian protein siderocalin (Scn) (Fig. 2a)^14,15^. This work has provided insight into the biological coordination of transplutonium elements while also offering new, qualitative information on metal-ligand covalency as protein recognition changes the electrostatic character of bonds in the actinide small molecule complexes. Beyond Scn, several endogenous proteins, including the iron transporter transferrin, are capable of directly binding to late actinides. Leveraging the presence of amino acid chromophores in the protein scaffold, which can act as sensitizing antennae, one can study metal-ligand coordination via time-resolved luminescence spectroscopy methods (Fig. 2b)^16^. Of note, a substantial advantage of using larger biomolecules for these structural and spectroscopic studies is the small amount of late actinide needed (ng to µg quantities for luminescence sensitization and protein crystallization, respectively).

**Fig. 2:**
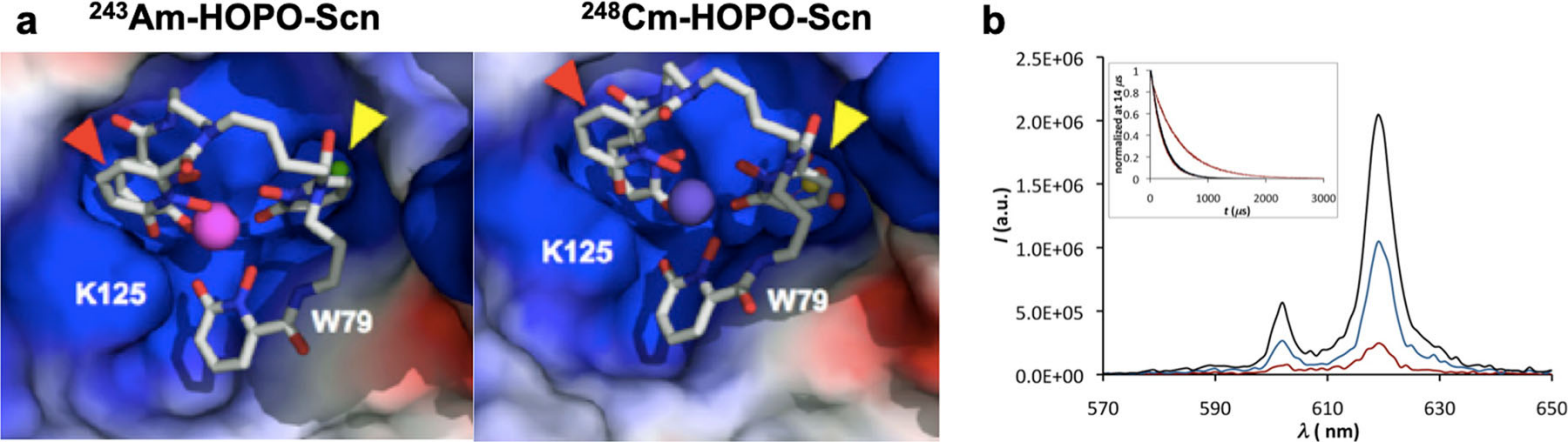
Alternative techniques for probing structural features of transplutonium complexes. **a** Macromolecular crystallographic analyses of the binding of chelated ^243^Am^III^ and ^248^Cm^III^ by siderocalin. The protein is shown in a molecular surface representation, colored by electrostatic potential (red = negative, blue = positive), the chelating group is shown in capped stick representation, colored by atom type (carbon: gray, nitrogen: blue, and oxygen: red), and the metal is shown as a colored sphere. Adapted with permission from ref. ^14^. Copyright 2015 National Academy of Sciences. **b** Luminescence spectra of solutions of Cm-transferrin in 100 mM NH_4_CO_3_ at pH 7.4. The inset shows lifetime measurements for all Cm-transferrin species upon protein excitation at 280 nm. Reproduced with permission from ref. ^16^. Copyright 2013 American Chemical Society.

Interpretation of experimental results indicating covalency in late actinide-ligand bonding is only made possible by concomitant theoretical analysis. This is best exemplified by recent work from Yang, Shafer, and co-workers who theoretically explored the covalency in Bk- and Cf-dipicolinates that had been reported by Albrecht-Schmitt (Fig. 3a)^10,11,17^. Density functional theory calculations did predict a decrease in 5*f*-orbital energy as one moves across late actinides for dipicolinate complexes; however, this meant covalency was driven by energy degeneracy (Fig. 3b)^17^, rather than metal-ligand orbital overlap, following principles developed and characterized, mostly by ligand K-edge X-ray absorption spectroscopy, in multiple early actinide systems^18^. Energy degeneracy driven binding is relevant in this part of the periodic table since decreasing 5*f*-orbital energetics across the series bring these orbitals into energy degeneracy with many common organic ligands, yet this framework for describing late actinide bonding does not account for spin-orbit coupling, as changes across the series from Russell-Saunders to *j–j* coupling schemes may impact transplutonium-ligand interactions.

**Fig. 3:**
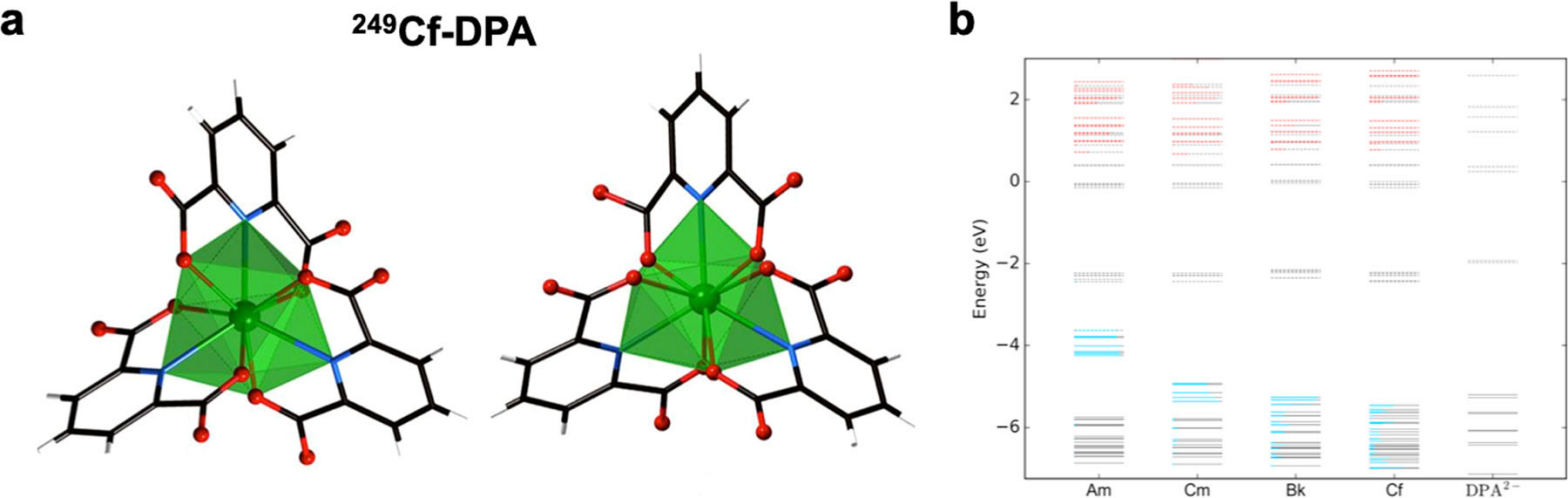
Probing late An^III^ covalency via DFT calculations. **a** Polyhedral view of enantiomers of Cf^III^(DPA)_3_^3−^ complex, which are representative of transplutonium-DPA structural results. Adapted with permission from ref. ^10^. Copyright 2015 Springer Nature. **b** Molecular orbital diagram of the An^III^(DPA)_3_^3−^ complexes (where An=Am, Cm, Bk, Cf) as well as ligand DPA^2−^. Blue and red colors represent An 5*f* and 6*d* atomic orbital contribution to the canonical molecular orbital, respectively. The length of the red or blue line is proportional to their contribution (percentage) in the canonical molecular orbital. Dashed lines represent unoccupied orbitals, and solid lines represent occupied orbitals. Reproduced with permission from ref. ^17^. Copyright 2017 American Chemical Society.

## Outlook

The development of transplutonium coordination chemistry is still nascent and determining the role of 5*f* orbitals in bonding and structure-property relationships is an area with significant growth potential within fundamental actinide science. As our understanding of covalency and bonding heterogeneities improves, it is possible to envision developing a general strategy for controlling structure and bonding within late actinide compounds, in the same way transition metal chemists use ligand and crystal fields to synthesize materials with tailored properties. Achieving this aim will require increased synthetic work in transplutonium coordination chemistry, which can be facilitated by continuing to expand isotope availability, as well as introduction of new characterization techniques such as magnetic circular dichroism (MCD) and electron paramagnetic resonance (EPR) spectroscopy to the late actinides. Harnessing this improved knowledge of covalency and bonding heterogeneities in transplutonium coordination chemistry has the potential to not only solve pressing needs in energy generation and waste management or to continue the quest for new elements, but also for new applications that will address pivotal contemporary issues. A prime example would be to leverage the large magnetic anisotropies and covalent bonding character observed in late actinide compounds^19^ for the development of new transplutonium materials with long spin-lattice relaxation times and enhanced qubit coherence times^20^, leading to the next-generation of quantum computing devices.
